# Chamber’s Case of Neuralgia Uteri

**Published:** 1850-09

**Authors:** E. D. Chambers

**Affiliations:** New Lebanon, Ind.


### ARTICLE VI.

*A Case of Misplaced Intermittent, or Neuralgia of the Uterus.* By E. D. CHAMBERS, M. D., of New Lebanon, Ind.

Mrs. R. æt. 19, a small, slender lady, was suddenly attacked on the 26th of April, 1849, with slight chilly sensations, nausea, headache, and excruciating pain in the region of the uterus. This latter symptom was periodical, alternating with intervals of total exemption from pain, and was so severe as to cause the patient to cry aloud.

In about one hour after her attack I visited her, and, on inquiry, was informed that about the same hour on the preceding day, the patient complained of feeling unwell, and suffered from uneasiness in the hypogastric region. In the course of an hour or two, the unpleasant symptoms entirely subsided, leaving the patient in her usual health, up to the time when I was summoned to attend her.

At my first visit I was unable to decide satisfactorily as to the exact character of the case, as, although in active practice for the last ten years, I had never before seen one similar. Nevertheless, I was not ignorant in regard to masked intermittents, or, as Eberle calls them, *Febres Intermittentes Larvatæ,* which so frequently attack the inhabitants of the Wabash valley, and thought it possible this might be one of the forms assumed by this Protean malady. I prescribed 15 grs. Hydrarg. Cum Creta and 10 grs. Pulv. Ipecac. Comp., and directed the application of hot flannel to the hypogastrium.

On the following morning the patient was free from any unpleasant symptons, but at 4 o'clock, P. M., she was again violently attacked. She took a grain and a-half of Opium, with ten grs. of Camphor, which afforded partial relief. Being now satisfied that her disease was of malarious origin, and that to be treated successfully, it must be regarded as a Quotidian Ague, I prescribed 20 grs. Quinine, and 15 grs.

Pulv. Ipecac. Comp. to be divided into four powders; one to be taken every three hours; commencing twelve hours previous to the expected attack. Since that date, she has been rid of the diurnal visits of her tormentor.

Except to show the close connection of malaria with the development of the diseases of the Wabash Valley, there is no remarkable interest attached to the above case. It may be remarked, however, that it serves to illustrate the truth of the remarks of Dr. Howland, in Vol. II. No. 1, of this Journal.

